# Ecologic Features of Plague Outbreak Areas, Democratic Republic of the Congo, 2004–2014

**DOI:** 10.3201/eid2402.160122

**Published:** 2018-02

**Authors:** Aaron Aruna Abedi, Jean-Christophe Shako, Jean Gaudart, Bertrand Sudre, Benoit Kebela Ilunga, Stomy Karhemere Bi Shamamba, Georges Diatta, Bernard Davoust, Jean-Jacques Muyembe Tamfum, Renaud Piarroux, Martine Piarroux

**Affiliations:** Ministry of Health, Kinshasa, Democratic Republic of the Congo (A.A. Abedi, B.K. Ilunga);; Plague Reference Laboratory, Bunia, Democratic Republic of the Congo (J.-C. Shako);; Aix Marseille University, INSERM, IRD, SESSTIM, Marseille, France (J. Gaudart);; UMR 6249 Chrono-environment CNRS/INRA/UFC, Franche-Comté University, Besançon, France (B. Sudre);; National Institute of Biomedical Research, Kinshasa (S.K.B. Shamamba, J.-J.M. Tamfum);; Aix Marseille University, CNRS, IRD, INSERM, URMITE, Dakar, Senegal (G. Diatta);; Aix Marseille University, CNRS, IRD, INSERM, URMITE, Marseille (B. Davoust);; Pierre and Marie Curie University, INSERM, IPLESP, Paris, France (R. Piarroux, M. Piarroux)

**Keywords:** Plague, epidemiology, Democratic Republic of the Congo, risk factors, environment, Yersinia pestis, bacteria, ecology, zoonoses

## Abstract

During 2004–2014, the Democratic Republic of the Congo (DRC) declared 54% of plague cases worldwide. Using national data, we characterized the epidemiology of human plague in DRC for this period. All 4,630 suspected human plague cases and 349 deaths recorded in DRC came from Orientale Province. Pneumonic plague cases (8.8% of total) occurred during 2 major outbreaks in mining camps in the equatorial forest, and some limited outbreaks occurred in the Ituri highlands. Epidemics originated in 5 health zones clustered in Ituri, where sporadic bubonic cases were recorded throughout every year. Classification and regression tree characterized this cluster by the dominance of ecosystem 40 (mountain tropical climate). In conclusion, a small, stable, endemic focus of plague in the highlands of the Ituri tropical region persisted, acting as a source of outbreaks in DRC.

Plague is a zoonotic disease caused by the gram-negative bacterium *Yersinia pestis* (*1*). According to World Health Organization (WHO) reports published in 2009 (*2*) and 2016 (*3*), >95% of the 15,396 cases reported worldwide during 2004–2014 occurred in Africa, especially in the Democratic Republic of the Congo (DRC, 8,379 [54%] cases); Madagascar (5,583 [36%] cases), Uganda (436 [3%] cases); and Tanzania (191 [1%] cases).

In DRC, plague was first reported in 1928, where J. Winderickx confirmed plague cases in Ituri (Orientale Province), near Lake Albert (*4*). In 1938, a second focus was discovered near Lake Edward (currently in North Kivu) (*5*), but no case has been reported there since 1967 (*6*). Before the 1950s, plague vaccination campaigns and rodent and vector control activities were conducted in both foci (*4*). Before the 1950s, the total number of notified cases remained low (Figure 1). Control programs then progressively collapsed, and the number of suspected cases notified to WHO dramatically increased in Orientale Province, peaking at 2,000 in 2006 (Figure 1).

**Figure 1:**
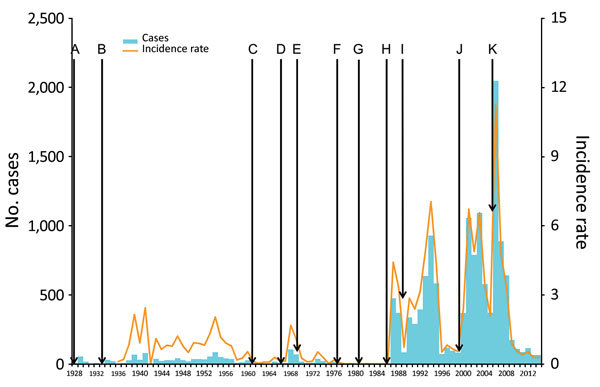
Timeline of plague cases, Orientale Province, Democratic Republic of the Congo, 1928–2014 (*2*,*4*–*7*). A) 1928: Detection of the first cases of plague in Ituri. B) 1933: First epidemiologic studies on plague. C) 1960: Independence of DRC, followed by the departure of expatriates dedicated to the fight against the plague. D) 1966: Armed conflicts in Ituri. E) 1968: End of postindependence conflicts. F) 1975: Surveillance and control assigned to the Ministry of Environment. G) 1979: Dereliction of control and reporting activities in Ituri. H) 1984: Control activities assigned to the Ministry of Health. I) 1987: First large epidemic episode, mostly in Ituri. J) 1996: Beginning of armed conflicts: first Congo war (1996), second Congo war (1999), and Ituri conflict (2003). K) 2003: Weakening of armed conflicts. Population data for former Kivu (corresponding to Maniema, North Kivu, and South Kivu) and Haut-Zaire (corresponding to Orientale Province) were calculated by smoothing data between the years with a known estimate (1947, 1955, 1975, 1984, 2000). Incidence is per 100,000 population.

Despite the high number of reported cases in DRC since the 1990s, almost no scientific reports have been published about plague foci in DRC. The 2 exceptions are an article describing the plague outbreak in Zobia (Ganga health zone [HZ]) in 2005 (*8*) and another about the laboratory confirmation of *Y. pestis* during 2 outbreaks (the previous outbreak in Zobia and another in Bole Bole, Wamba HZ, in 2006) (*9*). The recent events related to the Ebola virus disease outbreak in West Africa demonstrate the importance of remaining vigilant about highly virulent diseases still exhibiting a major epidemic potential (*10*), such as plague in DRC.

In this study, we aimed to describe the epidemiologic and ecologic characteristics of human plague during 2004–2014 in DRC. We also looked for spatially and temporally grouped cases (i.e., clusters).

## Materials and Methods

### Study Setting

**Figure 2:**
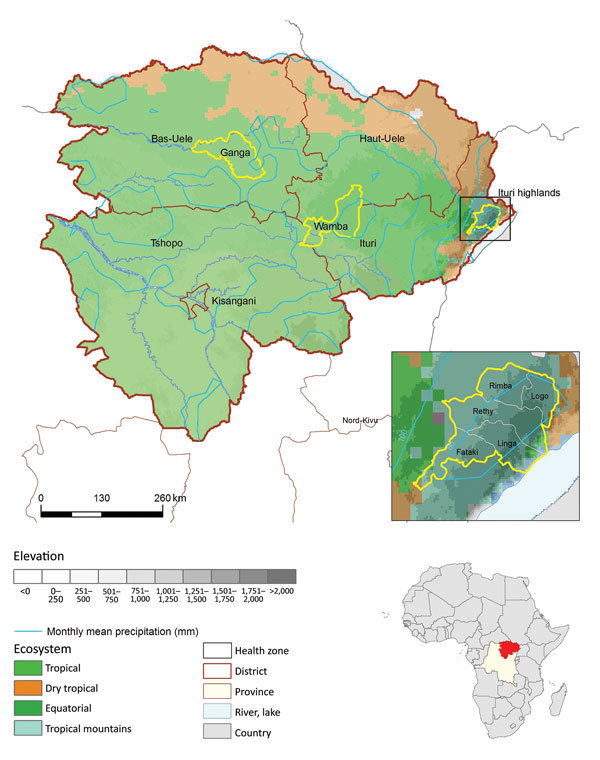
Location of spatial clusters of bubonic and pneumonic plague, Orientale Province, Democratic Republic of the Congo, 2004–2014. Yellow circles indicate clusters of health zones determined by spatial scan analysis. p values were <0.001, except for Wamba (p = 0.053). First inset shows the Ituri cluster constrained by frontiers; Oliveira F was 1 for Linga, Logo, Rethy, and Rimba and 0.69 for Fataki. Second inset shows location of DRC in Africa. The 4 ecosystems follow those described at http://www.fao.org/ag/AGAInfo/programmes/documents/livatl2/Ecosystems.htm (Technical Appendix Table).

We conducted the study in the former Orientale Province, located in northeastern DRC, the only place in DRC where plague was observed during the study period (Figure 2). At that time, the province (500,000 km^2^; mean population for the period 9.5 million inhabitants) was subdivided into 5 health districts (corresponding to the present eponym 4 provinces): Tshopo, Ituri, Haut-Uele, Bas-Uele, and Kisangani (currently included in Tshopo). The central and western parts of the province were covered by dense and humid tropical forests ranging from 200 m to 500 m in elevation (altitude). The north was covered by savanna vegetation and the east by savanna and crops; elevations ranged from 1,000 m in the plateau to >2,500 m in the mountains adjacent to Lake Albert (*11*). The province generally had an equatorial climate. Precipitation was abundant, ranging from 80 mm per month in Ituri to 200 mm close to the equator. In the eastern highlands, climate was cooler. The north and northeast experienced less rain and had a short dry season (December–February). The heaviest rains usually occurred in October and early November. Fishing, hunting, artisanal mining, and local trade were the main economic activities of the former Orientale Province (*12*), but in Ituri, residents made a living mainly from farming. During 1997–2003, Ituri was ravaged by armed conflicts that led to thousands of deaths, population impoverishment, and collapse of the healthcare system. The former Orientale Province population comprised 75.5% impoverished persons living in unsanitary conditions (*11*).

### Data Collection

In DRC, an Integrated Disease Surveillance and Response network surveyed 15 infectious diseases, including plague. Human plague data were collected every epidemiologic week at the HZ level, that is, the fourth health administrative level (the 5 nested health administrative units are State/Province/Health District/HZ/Health Area). The Ministry of Health provided more detailed databases for the initial period of Ganga epidemics in 2005 and Logo epidemics in 2014.

### Case Definition

The Ministry of Health provided a database of suspected plague cases; data were consolidated and completed with different patient line-listings and investigation reports performed during outbreaks. Suspected plague cases were defined according to WHO standard protocols (*13*). Cases were characterized by rapid onset of fever, chills, headache, severe malaise, prostration, with extreme painful swelling of lymph nodes (i.e., buboes) (bubonic form) or cough with blood-stained sputum, chest pain, and difficult breathing (pneumonic form). 

All suspected bubonic cases were kept in the database. Because the clinical definition of pneumonic plague is poorly specific, the Ministry of Health provides more accurate definitions during pneumonic outbreaks that account for the specific context (*14*,*15*). Pneumonic plague outbreaks have high case-fatality rates (CFRs) in developing countries, reaching almost 100% in the absence of proper patient care (*7*). Taking into account the observations of Neerinckx et al. (*7*) regarding CFRs, we applied a restrictive definition of suspected pneumonic plague outbreaks, discarding suspected pneumonic plague cases associated with low CFR (i.e., <5%), and without any biological confirmation. Implausible reductions in CFR, to <2%, were also observed at the end of plague-confirmed outbreaks. In 2004, for Ganga HZ, Bertherat et al. (*16*) proved that many of these cases resulted from other diseases, such as leptospirosis. We also discarded such cases from the database because, despite the presence of external medical teams assisting local response, we could not identify any confirmation of any pneumonic plague cases during postoutbreak periods.

An outbreak of pneumonic plague is defined as the presence of a single confirmed case of pneumonic plague. During outbreaks, suspected cases were confirmed by culture or by detection of *Y. pestis* F1 antigen in bubo aspirates using the rapid diagnostic test (RDT; Institut Pasteur, Antananarivo, Madagascar) or staining sputum smears (Gram and Wayson staining) according to standard protocols (*17*).

### Population and Environmental Data

We obtained HZ-level population data. In 2009, the Ministry of Health calculated HZ populations using data from the Expanded Programme on Immunization and from the Leprosy Elimination Program. We adjusted these data for the other years with the annual rate of natural growth (2.5%) (*18*). We also collected HZ-level environmental co-factors and retained the following co-factors for environmental analyses: elevation, land cover, precipitation levels, and climate type. Elevation data were derived from the Shuttle Radar Topography Mission (http://srtm.usgs.gov/) and monthly precipitation data retrieved from the TRMM (Tropical Rainfall Measure Mission) 3B43 version 7 (http://disc.sci.gsfc.nasa.gov/precipitation/documentation/TRMM_README/TRMM_3B43_readme.shtml). Land cover data were extracted from MODIS Yearly 12Q1 (Moderate Resolution Imaging Spectroradiometer, https://lpdaac.usgs.gov/dataset_discovery/modis/modis_products_table/mcd12q1). We summarized climate type ecoclimatic zones as defined on the website of the Food and Agriculture Organization of the United Nations (http://www.fao.org/ag/AGAInfo/programmes/documents/livatl2/africaezmaps.htm). These ecosystems are built incorporating repeated measures of NDVI (Normalized Difference Vegetation Index), temperature, rainfall, length of growing period, and elevation. Four ecosystems are present in Orientale Province: ecosystems 32, 33, 38, and 40, presented here as tropical, dry tropical, equatorial, and tropical mountain, respectively (Figure 2). Precise descriptions of these zones are available at http://www.fao.org/ag/AGAInfo/programmes/documents/livatl2/Ecosystems.htm and http://www.fao.org/ag/AGAInfo/programmes/documents/livatl2/afeztables.htm. 

We extracted and analyzed geographic data at the HZ level with ARCGIS 9.3 (ESRI, Redlands, CA, USA), using mean value and SD for elevation and precipitation and calculating the HZ proportion covered by each land cover or ecosystem type. We extracted the HZ map from the Health Mapper (*19*).

### Statistical Methods

Spatial case clusters were groups of contiguous HZs with significantly more suspected plague cases than the remaining HZs. To investigate clusters, we analyzed the number of cases in each HZ reported during January 2004–December 2014 using spatial scan statistics in SaTScan software (*20*,*21*). This approach systematically moves an elliptic scanning window of increasing diameters over the study region. For every diameter, it compares the observed case number inside the window with what would be expected over a random Poisson distribution of cases (*22*). The maximum allowed cluster size corresponded to 15% of the population of Orientale Province. We computed the Oliveira F coefficient for every HZ located in a likely cluster and obtained the statistical significance for each cluster with 999 Monte Carlo hypothesis testing, with a level of significance at 0.05 (*22*).

We assessed seasonal characteristics of case time series using seasonal trend decomposition based on local regression (*23*). We analyzed environmental co-factors using generalized additive models applied to a quasi-Poisson distribution, accounting for overdispersion, with log of population as offset and geographic coordinates as bivariate spline smoothing (*24*). For multivariate analyses, we assessed environmental patterns using the classification and regression tree method (*25*). This approach retains only the main co-factors among the collinear factors, thereby generating a tree in which the terminal nodes represent classes of HZs with common characteristics. Incidence ratios between classes issued from classification and regression tree analysis were estimated using the generalized additive model. Statistical analyses were provided by using R software version 3.1.3 (The R Foundation for Statistical Computing, Vienna, Austria).

## Results

### Global Morbidity and Mortality

**Figure 3:**
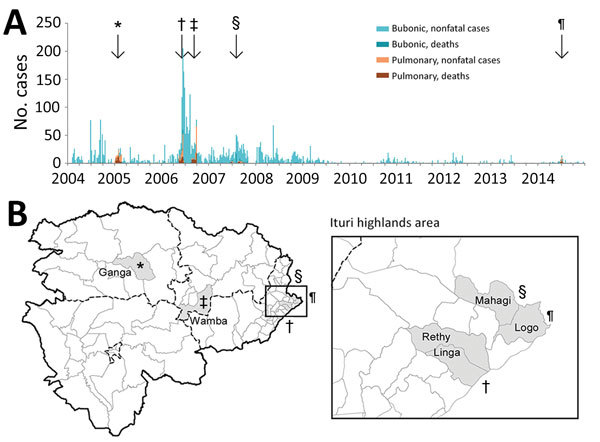
Temporal distribution of bubonic and pneumonic plague (A) and location of pneumonic plague outbreaks (B), Orientale Province, Democratic Republic of the Congo, 2004–2014. Five episodes of pneumonic plague outbreaks were observed; *, Ganga, 2005; †, Rethy and Linga, 2006; ‡, Wamba, 2006; §, Mahagi+Logo, 2007; ¶, Logo, 2014.. Ganga and Wamba experienced pneumonic plague only, after an increase of cases in the highlands of Ituri (enlarged area in B). Linga, Rethy, Mahagi, and Logo report bubonic plague all year but experienced outbreaks of pneumonic plague in 2006, 2007, and 2014.

During 2004–2014, a total of 4,630 suspected plague cases and 349 deaths (CFR 7.54%) were recorded in Orientale Province, DRC (Figure 3). These findings differ from WHO records (8,379 suspected cases and 464 deaths) and from the Ministry of Health database (5,153 suspected cases and 325 deaths) because we used a more restrictive definition of pneumonic plague and completed the database with line listings. Of these 4,630 cases, pneumonic plague accounted for 406 (8.8%) cases and 174 deaths (CFR 42.9%). Five pneumonic plague outbreaks were laboratory confirmed.

### Spatiotemporal Distribution of Human Plague

Spatial scan analysis showed that almost all suspected plague cases were recorded in 3 clusters: Ganga HZ, Wamba HZ (both located in equatorial forest lowlands), and 5 HZs in the Ituri highlands (Fataki, Linga, Logo, Rethy, and Rimba) (Figure 2). Results for Ganga and Ituri clusters were statistically significant (p<0.05), but the result for Wamba was not (p = 0.053). Wamba and Ganga experienced pneumonic plague epidemics; Ituri highlands recorded almost all bubonic cases.

### Bubonic Plague

**Figure 4:**
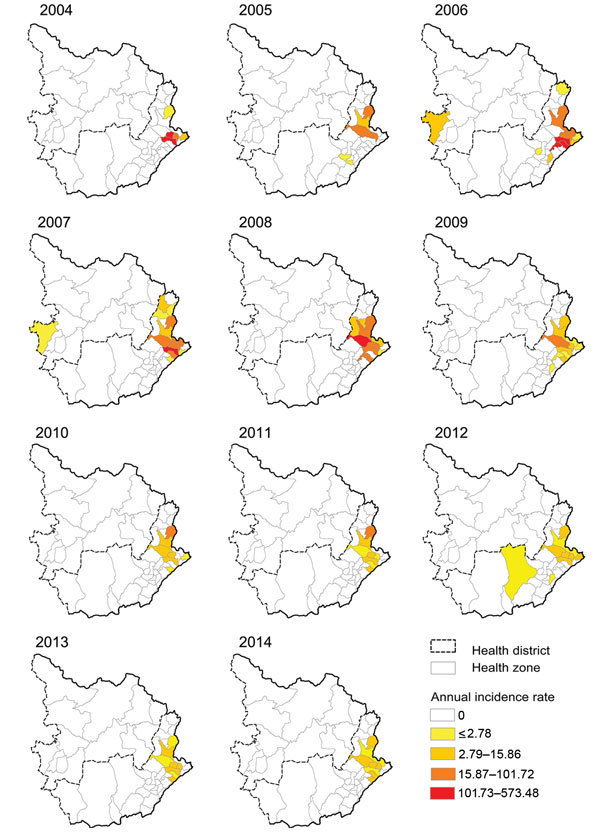
Yearly distribution of bubonic plague in Ituri and Haut-Uele districts, Orientale Province, Democratic Republic of the Congo, 2004–2014. The 2 eastern districts of Orientale Province (Ituri in the south and Haut-Uele in the north) were the only districts reporting bubonic plague during the study period. Highlands of Ituri had suspected cases every year. Incidence is per 100,000 population.

During 2004–2014, among the 4,224 cases and 175 deaths resulting from bubonic plague, 3,369 (79.8%) cases and 127 (72.6%) deaths were reported in the 5 HZs in Ituri highlands (Figure 4). Because of security concerns, bubonic plague was rarely laboratory confirmed. Before 2008, RDTs were distributed (thanks to Institut Pasteur of Madagascar) and used in Rethy, Linga, and Rimba. During 2007–2008, a total of 99 of 201 RDTs were positive. After 2008, the remaining RDTs were saved for outbreak investigations. 

**Figure 5:**
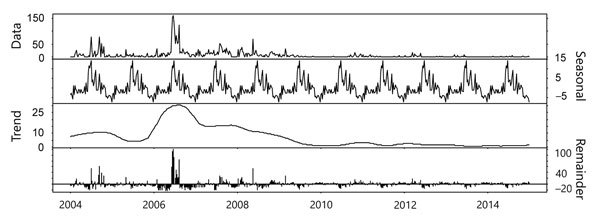
Time series decomposition using LOESS regression for bubonic plague, Orientale Province, Democratic Republic of the Congo, 2004–2014. Plague did not vary seasonally. The trend was decreasing after 2006. Remainder (residuals) explained 63% of model variance, trend 22% and seasonality 8% only.

In Ituri, bubonic plague cases were regularly notified, with seasonality explaining only 8% of the bubonic case time series variance (Figure 5). In 2007 and 2014, an increase in bubonic cases in Ituri highlands was followed by 2 smaller outbreaks of pneumonic cases. A field assessment in this area in April 2010 by several authors (A.A.A., J.-C.S., S.K., B.S., G.D., B.D., R.P.) highlighted various factors facilitating plague transmission (*26*,*27*). In particular, crops replaced cattle farming. Because of frequent thefts, seeds and food were stored in houses instead of within the traditional granaries built outside. The 50 dwellings visited were covered with vegetal roofs; >75% had >1 burrow on the ground; 90% of houses had 1 or 2 rooms; and 23 of 30 live rodents captured were caught inside the houses. Approximately 60% of inhabitants slept on the floor and were subject to flea bites.

### Pneumonic Plague

We identified 3 main outbreaks of pneumonic plague: 1 in 2005 and 2 in 2006. In 2007 and in 2014, two smaller outbreaks of pneumonic cases were recorded after an increase in bubonic cases in Ituri highlands (Figure 3).

#### Ganga HZ, 2005

Bertherat et al. (*8*) described the first recorded pneumonic plague outbreak in this region. The first cases were reported in January 2005 in a diamond mining camp near Zobia village in the Ganga HZ; at the same time, Ituri highlands were experiencing an increase in bubonic plague (Figure 3). Among the 89 (62%) patients with known occupations, 55 were miners; the male:female ratio was 4.5:1. The CFR ranged from 40% to 70% during the first weeks and suddenly decreased to <5% after the intervention of Médecins Sans Frontières (from Belgium). With the restrictive case definition, the overall CFR was 50/112 (44.6%). No case of bubonic plague was reported in the pneumonic plague–affected areas. As described by Bertherat et al. (*8*), 18 of 87 samples were positive by RDT and 32 by Wayson staining; cultures were negative.

#### Linga and Rethy HZs, 2006

A second plague outbreak occurred in May 2006 in Linga and Rethy HZs (Figure 3). These HZs regularly recorded sporadic cases of bubonic plague, in contrast with Ganga HZ. The index case-patient was diagnosed in Buba, Linga HZ. The patient first exhibited buboes, but septic shock developed rapidly, and the patient died within 2 days. His funeral rites enabled the disease to spread quickly. During epidemiologic weeks 20–25, a total of 119 cases of pneumonic plague were recorded; CFR was 36%, with a male:female ratio of 1.1:1. Fifteen of 56 samples were positive by RDT and 9 by Wayson staining; 7 *Y. pestis* isolates were cultured (*15*).

#### Wamba HZ, 2006

The third plague outbreak started in Wamba HZ in August 2006 (Figure 3) in a gold mining camp. The first identified case was a miner who had recently arrived; pneumonic plague developed on August 14, and he died 3 days later. Once again, the funeral rites enabled plague to quickly spread to the neighboring HZs of Pawa and Boma-Mangbetu. During the first weeks of the outbreak, the male:female ratio was 2.9:1 (*28*), and CFR was 44% (120 cases, 53 deaths) in these 3 HZs. Of 96 specimens, 23 tested positive by RDT and 2 by Wayson staining; 4 *Y. pestis* isolates were obtained (*9*).

#### Mahagi and Logo HZs, 2007

An outbreak of both bubonic (163 cases, 14 deaths; CFR 9%) and pneumonic (34 cases, 17 deaths; CFR 50%) plague was recorded in Mahagi and Logo HZs during weeks 15–44, 2007 (Figure 3). Seven samples were positive by RDT.

#### Logo HZ, 2014

The last recorded outbreak of pneumonic plague occurred in Logo HZ (Figure 3) during June 8–July 19, 2014. A total of 33 cases and 14 deaths were recorded, including 21 pneumonic cases (11 deaths, CFR 52%); 17 cases and 7 deaths were recorded in 3 villages only: Bika, Otha, and Jupathomba (male:female ratio 0.54:1). One patient was tested and confirmed positive by both RDT and culture.

### Environmental Analysis

We conducted univariate analysis for environmental factors (Table 1,Table 2). We retained variables at p<0.02 for multivariate analysis.

**Table 1:** Results of univariate analysis of environmental variables of bubonic plague, Orientale Province, Democratic Republic of the Congo, 2004–2014*

Variable	Estimate	SE	t value	p value
Ecosystem†				
Tropical	−0.06816	0.02595	−2.627	0.001
Dry tropical	−0.02004	0.004374	−4.582	<0.001
Tropical mountain	0.01751	0.003110	5.629	<0.001
Elevation, m, mean	0.004743	0.001066	4.449	<0.001
MODIS‡				
Water	−1.769	0.309	−5.726	<0.001
Evergreen broadleaf forest	−0.01278	0.007316	−1.748	0.086
Mixed forest	−0.4954	0.3251	−1.524	0.132
Closed shrublands	47.808	8.429	5.672	<0.001
Savannas	−2.0318	0.4889	−4.156	<0.001
Permanent wetlands	−0.50728	0.06268	−8.094	<0.001
Croplands	0.6011	0.1004	5.988	<0.001
Urban and built-up	−7.968	1.916	−4.159	<0.001
Rain accumulation, mm, TRMM3B43§				
Mean	0.008287	0.001643	5.044	<0.001
SD	−1.113	0.268	−4.150	<0.001

*Environmental variables with p<0.2 were kept for multivariate analysis. Negative estimates represent variables protective against plague; positive estimates represent variables increasing plague risk. †Ecoclimatic zones defined by the Food and Agriculture Organization of the United Nations (http://www.fao.org/ag/AGAInfo/programmes/documents/livatl2/africaezmaps.htm). These ecosystems are built incorporating repeated measures of Normalized Difference Vegetation Index, temperature, rainfall, length of growing period, and elevation. ‡Moderate Resolution Imaging Spectroradiometer (https://lpdaac.usgs.gov/dataset_discovery/modis/modis_products_table/mcd12q1). §Tropical Rainfall Measure Mission 3B43 version 7. (http://disc.sci.gsfc.nasa.gov/precipitation/documentation/TRMM_README/TRMM_3B43_readme.shtml).

**Table 2:** Results of univariate analysis of environmental variables of pneumonic plague, Orientale Province, Democratic Republic of the Congo, 2004–2014*

Variable	Estimate	SE	t value	p. value
Area	0.0005266	0.000142	3.709	<0.001
Ecosystem†				
Tropical	9.598	0.02713	353.8	<0.001
Dry tropical	−0.87697	0.01066	−82.29	<0.001
Equatorial	−5.7475	0.1096	−52.44	<0.001
Tropical mountain	0.5238	0.006693	78.26	<0.001
Elevation, m				
Mean	0.07305	0.001006	72.62	<0.001
SD	−0.1236	0.002218	−55.75	<0.001
MODIS‡				
Water	−217.2609	1.0011	−217.00	<0.001
Evergreen needleleaf forest	−18590.00	2116.00	−8.786	<0.001
Evergreen broadleaf forest	3.152	0.04331	72.78	<0.001
Deciduous broadleaf forest	908.7303	1.5165	599.2	<0.001
Mixed forest	25.4324	0.5472	46.48	<0.001
Closed shrublands	10081.9545	63.4111	159.0	<0.001
Woody savannas	3.9340	0.0521	75.51	<0.001
Savannas	−51.0000	1.1043	−46.18	<0.001
Grasslands	−139.8523	2.7516	−50.83	<0.001
Permanent wetlands	−40.8106	0.2148	−190.0	<0.001
Croplands	−7.9310	0.2875	−27.58	<0.001
Urban and built-up	96.1737	0.7640	125.9	<0.001
Cropland/natural vegetation	1.9249	0.0241	79.88	<0.001
Snow and ice	5639.5053	106.2298	53.09	<0.001
Barren/sparsely vegetated	17639.579	97.10	−181.7	<0.001
Mean rain accumulation, mm, TRMM3B43§	0.5343	0.005531	96.61	<0.001

*Environmental variables with p<0.2. These variables were kept for multivariate analysis. Negative estimates represent variables protective against plague, positive estimates represent variables increasing plague risk. †Ecoclimatic zones defined by the Food and Agriculture Organization of the United Nations (http://www.fao.org/ag/AGAInfo/programmes/documents/livatl2/africaezmaps.htm). These ecosystems are built incorporating repeated measures of Normalized Difference Vegetation Index, temperature, rainfall, length of growing period, and elevation. ‡Moderate Resolution Imaging Spectroradiometer (https://lpdaac.usgs.gov/dataset_discovery/modis/modis_products_table/mcd12q1). §Tropical Rainfall Measure Mission 3B43 version 7 (http://disc.sci.gsfc.nasa.gov/precipitation/documentation/TRMM_README/TRMM_3B43_readme.shtml).

**Figure 6:**
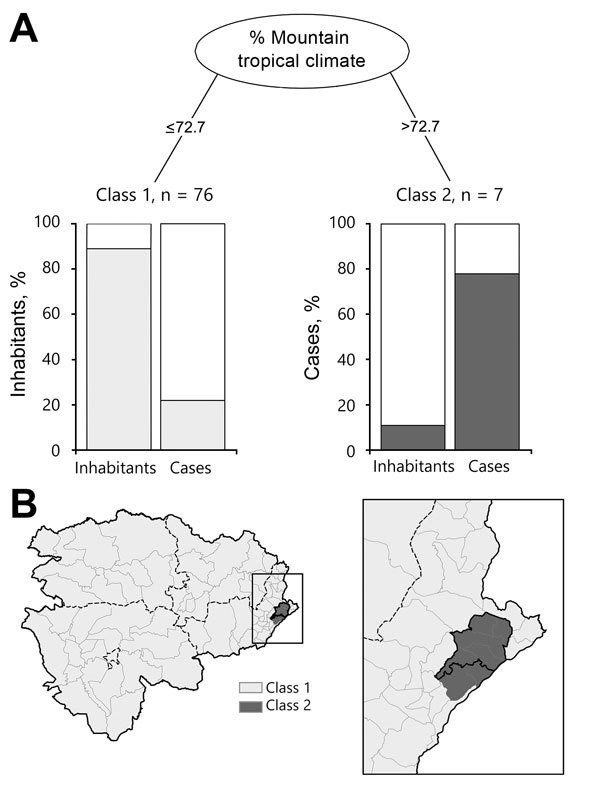
Classification of health zones according to environmental factors related to bubonic plague, Orientale Province, Democratic Republic of the Congo, 2004–2014. A) Classification and regression tree analysis of plague cases determined a significant (p = 0.015) high-risk class of 7 health zones (class 2). Health zones in class 2 have >72.7% of their territory in the mountain tropical climate. The increase in risk for class 2 compared with class 1 was not significant when analyzed with a generalized additive model (incidence rate 1.79; p = 0.14). B) Locations of class 2 zones within Orientale Province. Class 2 zones were grouped in the highlands of Ituri. The cluster determined by spatial scan statistics in the Ituri Highlands ("SaTScan cluster") was composed entirely of class 2 health zones.

Classification and regression tree showed 1 at-risk significant class (class 2, p = 0.014) for bubonic plague, encompassing 7 HZs, characterized by having >72.3% of their territory located in tropical mountain ecosystems (Figure 6). The same 7 HZs formed a nonsignificant at-risk class (class 2, p = 0.43) for pulmonary cases. The Ituri cluster is included in these 7 HZs. Compared with other ecosystems found in Orientale Province, the tropical mountain ecosystem is characterized by a lower maximum value and a later peak month for NDVI, lower average and minimum temperatures, less annual precipitation, shorter growing period, and higher elevation (Figure 2; Technical Appendix).

## Discussion

Our study shows that plague remains present in northeastern DRC, with yearlong bubonic cases in the mountains of Ituri and outbreaks of pneumonic cases. These outbreaks, for which the estimated CFRs range from 36% to 52%, occurred in the mountains of Ituri and in the equatorial forests of the province.

Access to health facilities was possible for only 37% of the population in Orientale Province (*12*), and data completeness often was low except during epidemics. Therefore, plague incidence could be underestimated, especially for sporadic cases. Conversely, overestimation of cases and underestimation of CFR are likely during large epidemics because of the low positive predictive value of the case definition and the lack of systematic biological confirmation of cases (*16*). To better estimate the reliability of diagnosis in the reported suspected plague cases, Neerinckx et al. (*7*) suggested comparing the reported CFR with the 12% CFR in Madagascar used as a reference for studies in Africa. Cases were overreported during the 2006 Wamba and Rethy outbreaks (*16*). We therefore used a restrictive definition of pulmonary cases according to CFR, discarding cases registered at the end of the epidemic, when CFR dropped dramatically.

In the future, overestimation might be resolved by improving laboratory capacities or using RDT directly in the field. However, such an approach requires a prepositioning of test dipsticks and staff training, supervision, and equipment to appropriately collect and process samples (*8*). These tasks are difficult to implement in the chaotic context of eastern DRC, especially in gold and diamond mines.

During the study period, cases and deaths were much higher than the 800 cases and 56 deaths recorded in the same area during 1928–1960 (*4*). The collapse of massive plague control activities might partially explain this difference (*29*).

Before 1950, the Natal multimammate mouse (*Mastomys natalensis*) was the more frequent commensal host for plague vectors in this region; identified vectors included *Xenopsylla cheopis* and *X. brasiliensis* fleas (*29*,*30*). Black rats (*Rattus rattus*), which live closer to humans, were first observed in the highlands in 1958 (*4*). The human flea (*Pulex irritans*), which enables interhuman transmission, was introduced in about the same period and became the most frequent indoor flea in Ituri (*29*,*30*). These introductions, associated with armed conflicts in 1997–2003 and other ecologic changes, probably facilitated plague transmission to humans.

The field assessment we performed in April 2010 highlighted at-risk contexts similar to what had already been observed in nearby Uganda, where rodents were abundant in households and residents kept crops inside their huts (*31*) and had no bedding material (*32*). In the mining camps in forest areas, explosive outbreaks of pulmonary plague in the absence of preceding sporadic cases might be due to an importation of the disease from an endemic area (*33*–*36*). These outbreaks were accelerated by funeral rites, when attendees embraced the corpse. Plague also might have spread because of the precarious life of miner populations settled in overcrowded camps.

Conversely, in the mountains of Ituri, a succession of outbreaks and perennial notification of sporadic bubonic cases since 1928 demonstrated a permanent transmission of the disease. This finding suggests circulation of the plague bacterium in >1 zoonotic host.

Human plague is an epiphenomenon of zoonotic plague, the incidence of which fluctuates in time and is modulated by numerous factors. In Tanzania, plague in rodents was linked to agricultural practices that lead to increased rodent populations but a lack of species diversity (*37*). In Madagascar, climate differently influenced plague transmission in rats (favored by urban areas, low temperature, and humidity) (*38*,*39*) and in wild rodents (favored by semiarid regions and the end of the dry season) (*40*). In Uganda, fewer fleas on rodents were observed during the dry season (*41*). Ituri is close to the equator (2°N), which can explain the lack of seasonality in human plague.

Plague in humans has been linked with behaviors and environmental hazards (*1*,*42*,*43*). In DRC, bubonic plague was associated with the tropical mountain ecosystem. This relationship should be interpreted cautiously, however, because all plague-endemic HZs are grouped in a single mountainous area. When a model based on the neighboring Uganda focus was applied, Ituri highlands were considered suitable for plague occurrence (*44*).

It would be interesting to study the Uganda focus and the adjacent Mahagi district as a single focus. Elevations, dry season temperatures, slopes, and landscapes differ slightly between this Mahagi–Uganda focus, which is located mostly in a dry tropical ecosystem, and the Ituri highland focus, which is even wetter and colder. In Uganda, the risk for plague also increased with wetness (*44*).

Identifying animal reservoirs or assessing genetic exchanges between *Y. pestis* populations in both foci could be of great interest. The old and possibly extinct focus near Lake Edward displays the same characteristics as the Ituri highlands. If security improves, it could also be useful to actively search for plague in rodents in this area.

Previous observations showed that the risk for plague tends to increase in highlands covered by savannas or meadows with a relatively dry climate (*32*,*45*). In Madagascar, the rural elevated districts act as permanent foci from which less favorable areas develop pulmonary epidemics (*40*). In DRC, the Ituri cluster, well summarized by a tropical mountain ecosystem, acted as a homeland for the plague. This region combines many of the previously described factors.

Because the Ituri focus is among the most active plague foci in the world and is probably at the origin of outbreaks of pneumonic plague that spread in the forest, extending the epidemiologic and ecologic studies of the plague in this focus is of paramount importance. As emphasized by Stenseth et al., “… plague should be taken much more seriously by the international community than appears to be the case” (*46*). The present-day priority should include establishment of a local and reactive surveillance system together with the improved rapid biologic confirmation of cases to earlier detect and better contain plague outbreaks (*47*).

Technical Appendix Description of 4 ecosystem classifications from the Food and Agriculture Organization of the United Nations.
