# Supplementary material for: Ecologic Features of Plague Outbreak Areas, Democratic Republic of the Congo, 2004–2014
Source: Emerg Infect Dis. 2018 Feb;24(2):210–20. doi: 10.3201/eid2402.160122 (PMC5782875; doi:10.3201/eid2402.160122)
Supplement: Technical Appendix — Description of 4 ecosystem classifications from the Food and Agriculture Organization of the United Nations. [file 16-0122-Techapp-s1.pdf]

Article DOI: http://dx.doi.org/10.3201/eid2402.160122

Ecologic Features of Plague Outbreak Areas, Democratic Republic of the Congo, 2004–2014

Technical Appendix

Technical Appendix Table. Partial reproduction of the table describing the 4 FAO ecosystems at http://www.fao.org/ag/AGAInfo/programmes/documents/livatl2/afeztabless.htm*

Ecosystem	Normalized Difference Vegetation Index				Temperature, °C					
(name)†	Average	Min	Max	PHS1	Average	Min	Max	LGP	Rain, mm‡	Elevation, m
32 (Tropical)	0.45	0.37	0.54	5.5	32.2	30.0	35.9	337	1,851	602
33 (Dry tropical)	0.40	0.23	0.53	6.8	35.1	30.6	43.7	268	1,668	772
38 (Equatorial)	0.44	0.32	0.54	2.4	32.6	30.4	35.2	335	1,784	531
40 (Tropical mountain)	0.40	0.28	0.51	7.9	31.0	25.5	38.8	239	1,427	2131

*See Figure 2. FAO, Food and Agriculture Organization of the United Nations; LGP, length of growing period (days); PHS1, the Fourier Phase 1 parameter.
†Eco-climatic zones defined by the FAO, http://www.fao.org/ag/AGAInfo/programmes/documents/livatl2/Ecosystems.htm.
‡Cumulative annual rainfall.
